# A quasi-experimental study of ethnic and gender bias in university grading

**DOI:** 10.1371/journal.pone.0254422

**Published:** 2021-07-22

**Authors:** Carina Saxlund Bischoff, Anders Ejrnæs, Olivier Rubin

**Affiliations:** Department of Social Sciences and Business, Roskilde University, Roskilde, Denmark; TED University, TURKEY

## Abstract

This paper contributes to the debate on race- and gender-based discrimination in grading. We apply a quasi-experimental research design exploiting a shift from open grading in 2018 (examinee’s name clearly visible on written assignments), to blind grading in 2019 (only student ID number visible). The analysis thus informs name-based stereotyping and discrimination, where student ethnicity and gender are derived from their names on written assignments. The case is a quantitative methods exam at Roskilde University (Denmark). We rely on OLS regression models with interaction terms to analyze whether blind grading has any impact on the relative grading differences between the sexes (female vs. male examinees) and/or between the two core ethnic groups (ethnic minorities vs. ethnic majority examinees). The results show no evidence of gender or ethnic bias based on names in the grading process. The results were validated by several checks for robustness. We argue that the weaker evidence of ethnic discrimination in grading vis-à-vis discrimination in employment and housing suggests the relevance of gauging the stakes involved in potentially discriminatory activities.

## Introduction

While there appears to be extensive evidence supporting a negative job market bias for ethnic minority-sounding names in general [cf. 1–6] and in Denmark in particular [7, 8], there is conflicting evidence in the academic literature as to whether ethnic and gender bias can be identified in grading. Some examiners might “punish” examinees with “ethnic” sounding names due to subconscious or conscious ethnic or religious discrimination. Others might “reward” the same students by giving them preferential treatment if they exceed the expectations that are set lower for them than for the ethnic majority. Importantly, both of these biases reflect some kind of ethnic stereotyping, and ethnic biases in grading that are rooted in ethnic stereotypes can therefore be both positive and negative. Some studies have found a negative bias against ethnic minorities in grading. Analyzing caste discrimination in India using an experimental design, Hanna and Linden (2009) found that teachers graded exams by students perceived as being lower caste slightly lower than exams by students perceived as being high caste. Interestingly, they found that the discrimination against low caste students was driven by lower-caste teachers, whereas higher-caste teachers did not appear to discriminate [9]. Botelho, Madeira, and Rangel (2015) found racial discrimination within racially integrated eighth grade public school math classrooms in Brazil. White students, the authors found, were less likely to be deemed non-competent (below passing grade) than their equally proficient and equivalently well-behaved black classmates [10]. In a European context, Sprietsma (2013) found that 88 primary school teachers in Germany graded what they believed to be assignments written by ethnic minority students slightly lower than identical assignments written by ethnic majority students [11]. Based on a similar experimental design, Hinnerich, Höglin, and Johannesson (2015) found a more sizeable and robust discriminatory effect in public upper secondary schools against students with foreign backgrounds in the grading of a Swedish national test [12]. Other studies, all based on European data, found limited evidence of bias against ethnic minorities. Riegle-Crumb and Humphries (2012) found no bias against ethnic minorities in how British math teachers perceive students once controlling for differences in grades and test scores [13]. Similarly, Aldrin (2017) relied on an experimental design of how 113 Swedish upper secondary school teachers assessed the same assignment, the only difference being whether an ethnic minority (Mohammed) or Swedish (Carl) name had been inserted mid-text, and she found no significant difference in the grading of the two groups of assignments [14]. Using a similar experimental research design, where Dutch teachers graded assignments that differed only with respect to whether the student name belonged to an ethnic minority or not, Van Ewijk (2011) also found that “teachers do not give lower grades for essays that were purportedly written by ethnic minority students than for the same essays when purportedly written by ethnic majority students” [15: 1055]. In terms of gender, most studies tend to find either no gender bias [cf. 9, 12, 16] or a grading bias against boys [for an overview, see 17, 18]. Other studies, however, found a slight bias against female students in the grading of a macroeconomics exam for an introductory course at Stockholm University [19] or evidence of stereotyping among U.S. high school teachers, where girls were believed to be worse at math than boys [13]. Thus, the direction and size of gender and ethnic grade bias remains an open question.

This study makes use of a quasi-experimental design, our hypothesis being that written examinations revealing the examinees’ names may bias grades through the examiners’ subconscious or conscious gender and ethnic stereotypes and discrimination. Exploiting a change from 2018 to 2019 in the anonymity of exam submission, we investigate whether knowledge of student identities affects grading. Thus, if grading is subject to gender and/or ethnic biases, we would assume: (i) that the grade differences between male and female examinees are less in 2019, where examinations were graded blindly, compared to 2018 and (ii) that the grade differences between ethnic minorities and majorities are reduced in 2019, where examinations were graded blindly, compared to 2018 and (iii) the extent/direction of the reduction in grade differences in 2019 compared to 2018 for all types of examinees (male/female; ethnic majority/minority) might depend on the examiner’s gender.

## Materials and methods

Roskilde University (RU) is a relatively small university (approx. 8,000 students) on the outskirts of Copenhagen. The collection of data was cleared by Roskilde University’s ethical and legal department on the condition that the publicly available data was anonymized. All participants in the study were informed of the purpose of the research and were given the opportunity to opt-out. None objected to the use of their data. Around 5–600 students enroll in the social science bachelor’s degree program each year, and one of the mandatory courses in this program is a quantitative methods course (fourth semester). The course consists of ten lectures together with exercises aimed at introducing students to basic statistical methods applied to social science themes. The course ends with a 3.5 hour in-house written exam that is subsequently graded by an internal examiner according to the Danish 7-point grading scale (-3, 00, 2 (pass), 4, 7, 10, 12). The social science bachelor’s degree program at RU consists of two parallel tracks: an English language track primarily for international students or students with limited command of Danish (20% of the students) and a Danish language track primarily for students who have graduated from a Danish upper secondary school (80%). Our study only includes data from the Danish track, as the courses run separately.

### Research design

We exploit several methodological advantages to construct our quasi-experimental research design.

In terms of data availability, the quantitative methods exam is one of the most comprehensive exams at RU: in the Danish language track, 428 written exams were graded in 2018, and 470 in 2019 (incl. both ordinary exams and reexaminations). This implies that we have sufficient data to make fairly robust inferences for the subsamples of interest for both years.

The study makes use of a unique quasi-experimental design made possible by a shift in university grading practices from open to blind in 2019. One of the natural sciences departments with a very low student/teacher ratio found it challenging that teachers consistently had to grade students with whom they were well acquainted. Consequently, with support from student representatives, they pushed for change. The matter was raised in the university’s central decision-making body for educational matters, where it was decided to transition to blind grading. The decision has been implemented somewhat unevenly by the administrative staff responsible for setting up the exams; to this day, several written exams within the department are still graded openly. Of critical importance for this study, the decision to adopt blind grading for the quantitative methods exam was not discussed with any of the staff involved in the quantitative methods exam in advance. From the perspective of these examiners, numbers simply replaced names in the 2019 assignments without any preceding discussion or notification. Thus, for the purpose of the study, the change of practice can be considered exogenous. Another advantage of the research design is the fact that name internalization prior to 2019 was high, as the examinee was to write their name in the header of every page of the exam. More importantly, examiners were given a grading template by the course convener, where the examiners were asked to write down the name of the individual examinee together with their scores and final grade. This grade was then assigned to the examinee’s name in the formal online grading system; in other words, the examiners could not avoid noticing the examinees’ names, which creates lush conditions for conscious or unconscious grade bias based on gender or ethnic stereotypes as deduced from the names. The exam format itself also creates ample opportunity for bias: The exam assignment consisted of around twenty sub-questions, each of which was scored by the examiner. These scores were then aggregated to a total score that formed the basis for the final grade. The questions consisted mainly of interpretive statistical questions, with answers extending beyond mere numerical values. They were asked to propose and critique survey questions, explain rationales for the chosen statistical tests, give interpretations of the results obtained and brief discussions of the policy implications. In short, the answers were not simply “true/false.” As the exam consisted of mostly interpretive statistical questions, there was ample scope for idiosyncratic judgements when scoring each sub-question. However, the questions did not invite the type of personal reflections or use of language that might reveal an examinee’s background. Finally, examiners are unlikely to associate names with actual students with whom they have interacted in class; while some of the examiners had also lectured the class, these lectures were conducted in huge lecture halls with hundreds of students. The subsequent exercises in smaller classes were largely left to junior instructors, who do not grade exams. Moreover, the exam papers were randomly assigned to examiners, making it highly unlikely that an examiner would be able to associate a particular name with a particular examinee.

One important assumption in this experimental design is that we assume that the relative qualifications between the major groups (based on male and female names as well as ethnic minority and majority names) remained constant from 2018 to 2019. In other words, the relative differences in the qualifications between the groups within the 2018 cohort of students are similar to the relative differences in the qualifications between the groups within the 2019 cohort. We cannot test this assumption directly, but a comparison of the two cohorts reveals that they are highly similar; first, the grade point averages of upper secondary school students admitted in the two cohorts were very similar: 7.74 for the 2018 cohort (admitted in 2016) and 7.82 for the 2019 cohort (admitted in 2017). Moreover, there was no discernable difference in the academic backgrounds of the students, and the gender ratios for the two years were very similar. While Danish universities do not collect enrollment data based on ethnicity, Statistics Denmark has found similar country-wide ratios of ethnic minorities enrolled in social science bachelor programs for the two years [20]. Moreover, the percentages of the total number of exam assignments (ordinary exam and reexaminations) with ethnic minority names (c.f. below) are very similar for the two years in question (14.3% and 15.7%). We therefore believe this is a reasonable assumption. The relative differences between the cohorts are likely to be fairly constant from one year to the next under the same admission procedures, university rules, and course requirements (see, however, Discussion for potential minor sources of bias)

#### Associating student names with gender and ethnicity

While the most obvious way to gauge an examinee’s ethnicity and gender in a written examination is by their name, more subtle cues can also play a role. It might thus be possible to infer the gender or ethnicity of the author of a written assignment indirectly from the theme of the assignment, the use of language, personal pronouns, and so forth [e.g., 21, 22]. For this exam, however, the latter type of inference is unlikely for two reasons: First, the students’ command of Danish is generally good. All of the ethnic minority students in the program have graduated from Danish upper secondary schools and have obtained the grades required for admission. Secondly, the exam in quantitative methods poses a fixed set of very specific questions related to statistical methods. The language used to answer such questions is typically succinct, and the exams do not include lengthy essay questions. Consequently, opportunities for making indirect inferences regarding the examinee’s identity based on language use are quite limited. We therefore find it reasonable to assume that the examinee identities (and any ensuing group-based stereotyping) would almost exclusively be deduced from the names provided on the assignment. Name-based stereotyping implies that examinees from ethnic minorities who have adopted names common to the ethnic majority will not be subject to discrimination. Likewise, a transgender person would be graded based on the name they write on the day of the exam, and thus potentially subject to gender-based stereotyping related to that name.

In order to associate the names with gender and ethnicity, the three authors independently coded the names in the dataset according to gender and ethnicity. Our profiles are very similar to the examiners in terms of shared educational background, being in the same median age bracket, all belonging to the ethnic majority, and having the same gender balance (two males, one female). The examiners were from RU (the exam uses internal assessment) and all belonged to the ethnic majority. Seven examiners (two females, five males) graded the assignments: two examiners graded only in 2018, two examiners only in 2019 and three examiners graded exams in both years. Thus, based on our similar backgrounds, we are likely to share the examiners’ gender and ethnic associations from the student names. Each of us coded the names independently. We assigned the value 1 if we perceived the students’ name as female and 0 if male. In addition, we assigned the value 1 for names associated with ethnic minority students and 0 for ethnic majority. Although it is unclear how insights into the purpose of the study would affect the assignment of gender and ethnicity, it is important to note that only the student names were visible to us and the grades were hidden. Each coder was to assign the values swiftly to get the immediate and intuitive association between student name and ethnicity/gender in much the same way as the examiners in the grading situation. It should be noted that in Denmark, as in most of Europe, ethnic minority names are typically distinct from ethnic majority names. Gender and ethnicity were coded for all names. The name‒gender/ethnicity association was very similar for all three coders (Cohen’s kappa for coding of ethnic names = 0.89; Cohen’s kappa for coding of gender names = 0.90), and there was only disagreement on about 7.2% of the cases for gender and 4.1% for ethnicity. The most variation across researchers was found in associating gender to names primarily belonging to ethnic minorities, which is to be expected when the researchers all belonged to the ethnic majority. In the subsequent analyses, we used the code that two of the three coders had assigned. However, we also conducted sensitivity calculations where we only included observations where there was full agreement on the ethnic or gender associations provoked by the names.

The cases are the written exams. As mentioned, there are 898 in total (428 with names on the exam in 2018; 479 blind in 2019). This number includes 725 exam papers from the ordinary exam and 174 at the re-examination. The re-examination includes exams from students who fail the first exam, who were ill, or who simply decided to postpone taking the exam. There is no risk of an examiner remembering a name from the ordinary exam and repeating bias at the reexamination, which would make the cases non-independent, because none of the examiners who graded the ordinary exam in 2018, where names appeared on the exam papers, also graded the reexamination. This means that we can safely regard the cases as independent with respect to grading bias.

## Results

135 assignments were coded as having ethnic minority names (61 in 2018, 73 in 2019), while 478 assignments were coded as having female names (220 in 2018, 258 in 2019).

Table 1 displays the descriptive statistics (average grade in 2018 was 4.2, and 5.2 in 2019). Although the exams contained the same number of questions with the same balance between purely statistical calculations and more interpretive answers, an obvious explanation for the higher average grade might be slightly easier questions in the 2019 exam. Some minor improvements have also been made to the course, however, most notably facilitating voluntary “study cafés,” which might have boosted student performance. The standard variation for both years was almost identical (4.15 in 2018, 4.10 in 2019), indicating that the distance between the high and low performers had not increased.

**Table 1:** Summary statistics.

Year	Coded identity	Mean	Std. Deviation	N	Mini- mum	Maxi- mum	Share of total (%)
**2018**	Ethnic majority	4.6	4.2	367	-3	12	86%
	Ethnic minority	1.9	2.8	61	0	10	14%
**2019**	Ethnic majority	5.7	4.1	396	0	12	84%
	Ethnic minority	2.5	3.2	74	0	10	16%
**2018**	Male	4.1	4.2	208	-3	12	49%
	Female	4.3	4.1	220	0	12	51%
**2019**	Male	5.0	4.0	212	0	12	45%
	Female	5.3	4.2	258	0	12	55%
**2018**	All	4.2	4.2	428	-3	12	100%
**2019**	All	5.2	4.1	470	0	12	100%

In 2018, the average grade of students with ethnic majority names was 4.55, compared to 1.85 for those with ethnic minority names. In 2019, the average grade was 5.66 for the former group of students and 2.49 for the latter group. In both years, these grade differences between ethnic majority and ethnic minority students are statistically significant.

In 2018, the average grade was 4.26 for students with female names and 4.06 for males. Again, the grades were higher across genders in 2019, where students with female-sounding names averaged a 5.33, whereas male-sounding names scored 4.96. The gender differences were not statistically significant in either year.

**Fig 1:**
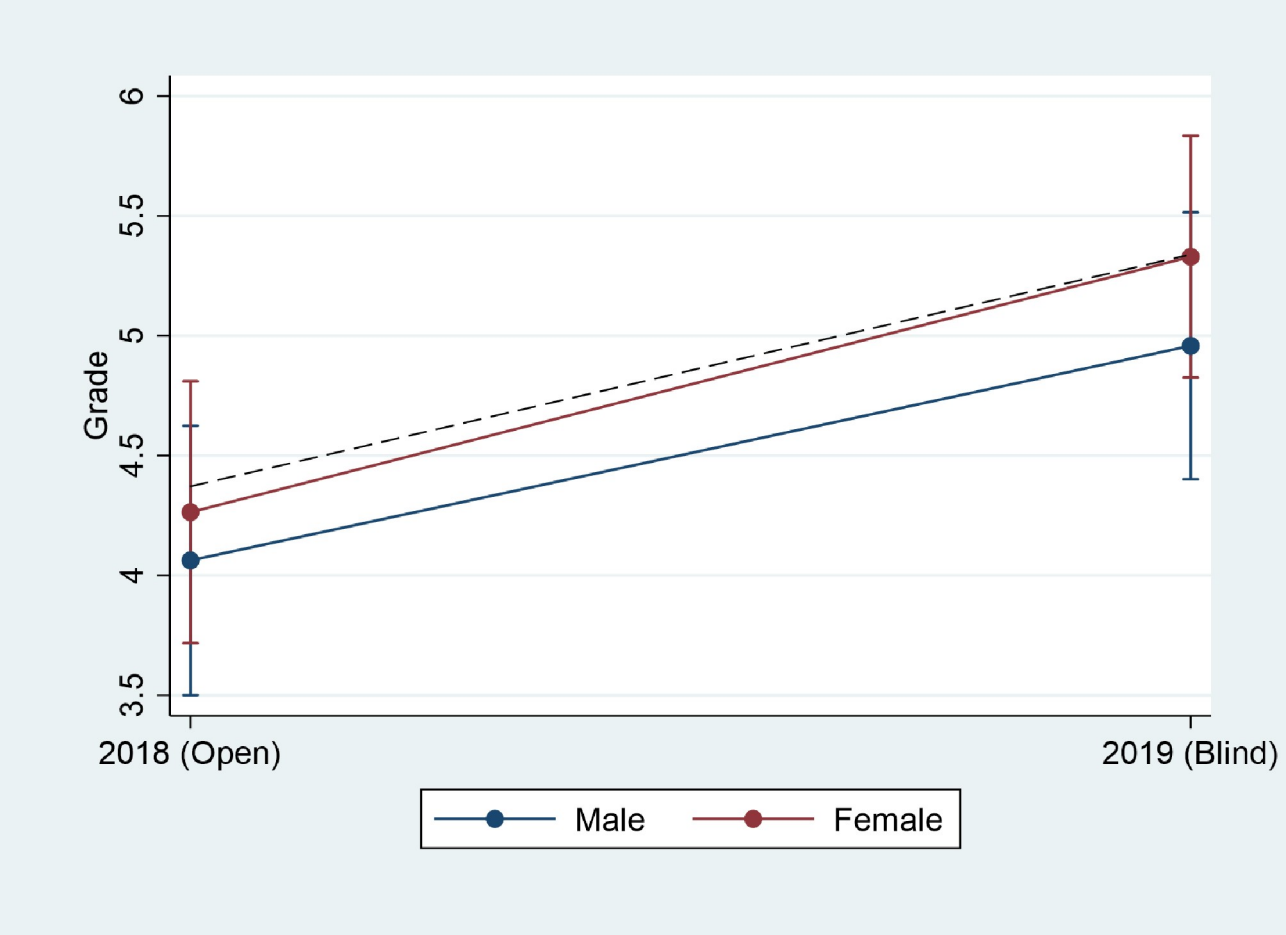
Gender bias. The dashed line represents the predicted grades of students with female names with no gender bias.

**Fig 2:**
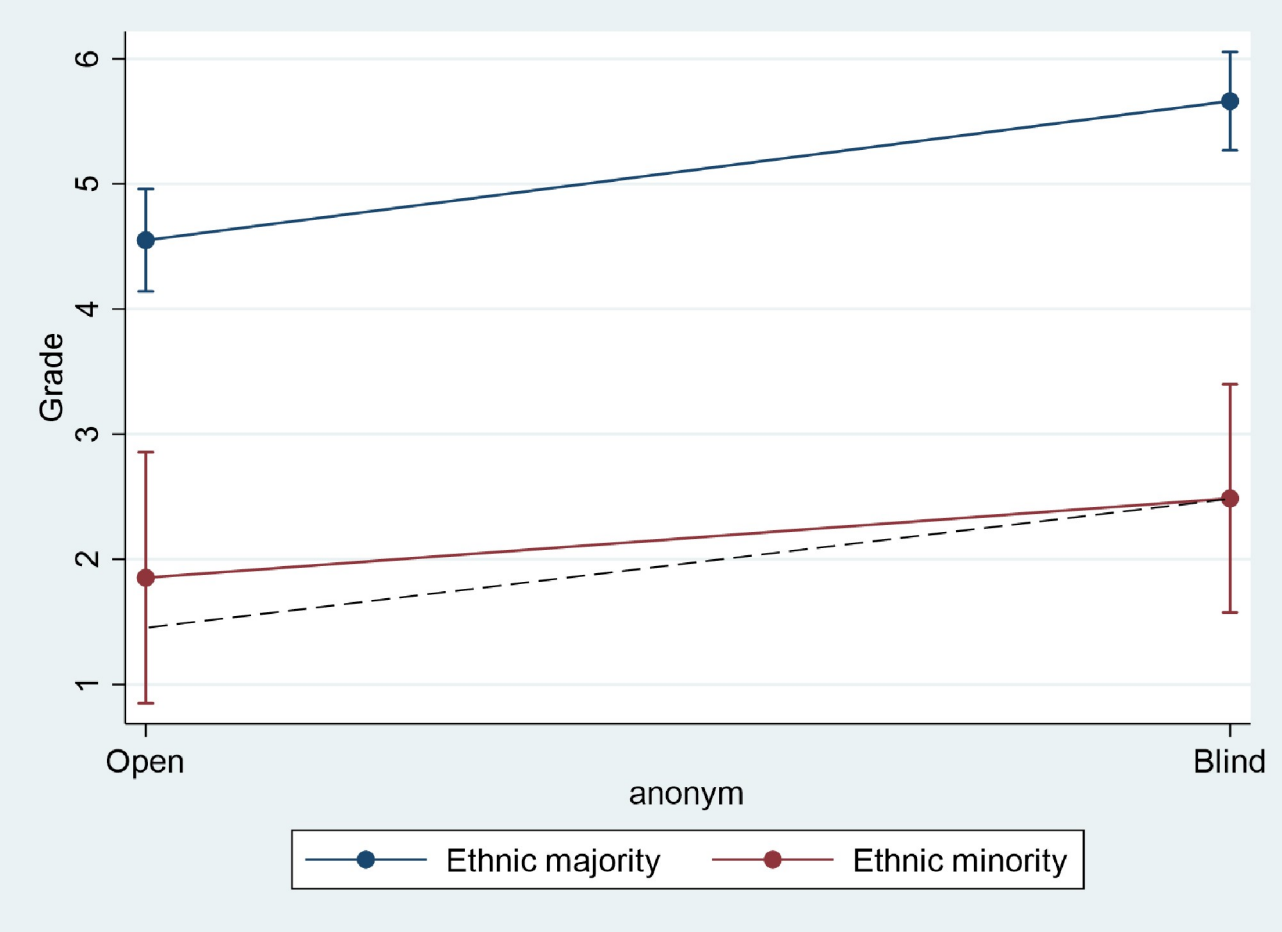
Ethnic bias. The dashed line represents the predicted grades of students with ethnic names with no ethnic bias.

Figs 1 and 2 illustrate the descriptive results by interacting the gender and ethnicity variables with the variable indicating whether the exam was blind or not. Fig 1 shows that female examinees received higher grades at the blind exam than expected. This is indicative of a slight, negative gender bias in the sample; that is, female examinees receive slightly lower grades when examiners can see their names. The result is not significant, however.

Fig 2 illustrates the interaction effect between the ethnicity and blind variables, revealing that ethnic minority students receive a lower grade in the blind exam than expected, which would indicate positive bias (i.e., that examiners tend to give slightly higher marks to examinees in the sample they believe belong to the ethnic minority). However, the slight bias in the sample is insignificant. The analysis therefore does not lend support to the hypothesis that examiners bias (either positively or negatively) grades for ethnic minorities.

The next step is to test for bias in a multivariate model with controls. In addition to anonymity, gender, and ethnicity, we also control for whether the exam was an ordinary exam or a reexamination. Those who sit for a reexamination were either ill at the time of the ordinary exam, decided not to attend, or attended and failed the ordinary exam. Not surprisingly, therefore, the average grade for the reexamination is typically lower than for the ordinary exam. The ordinary exam in 2018 resulted in a 4.32 average grade compared to 3.54 for the re-exam. In 2019, the average grade for the ordinary exam was 5.61 compared to 3.22 for the re-exam. This difference is statistically significant. While there is nothing to suggest that this difference would have any bearing on gender and ethnic bias, we nevertheless included the variable as a control in the analysis.

In order to identify discrimination based either on ethnicity or gender, we run two OLS regression analyses: one with an interaction term for open exam and gender, and one for open exam and ethnicity.

G = β0 + β1O +β2OR + β3F + β4E + β5(O*F)+ ε
G = β0 + β1O +β2OR + β3F + β4E + β5(O*E)+ ε

- G is the grade obtained for the quantitative methods exam.
- O is a binary variable. 1 represents students who took the open exam in 2018, 0 represents those who took the blind exam in 2019.
- OR Is a dummy variable indicating whether the student has taken the ordinary exam or the reexamination.
- F is a gender dummy variable. 1 represents examinees with female names, 0 represents examinees with male names.
- E is an ethnic dummy variable. 1 represents examinees with ethnic minority names, 0 represents examinees with ethnic majority names.

The interaction term coefficient indicates whether the grade differences between gender or ethnic categories differs between the open exam compared to the blind exam. If the coefficient for the interaction term is 0, then gender or ethnic differences remain constant between 2018 (open exam format) and 2019 (blind exam format).

Table 2 reports the results of the regression analysis where we test for gender and ethnic bias in grading. Model 1 is the basic model without the interaction terms; that is, it tests whether the variables gender, ethnicity, ordinary exams, and open exams are associated with grades. In line with the descriptive statistics reported above, the β1 estimate indicates that students obtained significantly higher grades in 2019—around one point higher on the scale on average. The β2 shows that students score more than one point higher at the ordinary exam than at the reexamination. In Model 1, the β3 estimate also reveals that students with ethnic minority-sounding names obtain lower grades than students with ethnic majority-sounding names (on average 2.76 points).

**Table 2:** Gender and ethnic based bias in grading.

OLS regression		Model 1			Model 2			Model 3		
			*Basic model*		*Gender bias*			*Ethnic bias*		
		B	SE	P value	B	SE	P value	B	SE	P value
β0	Intercept	**4.48**	0.37	0.00	**4.46**	0.39	0.00	**4.51**	0.38	0.00
β1	Open	**-1.02**	0.27	0.00	**-0.98**	0.39	0.01	**-1.07**	0.29	0.00
β2	Ordinary exam	**1.27**	0.34	0.00	**1.27**	0.34	0.00	**1.26**	0.34	0.00
β3	Female	**0.16**	0.27	0.55	**0.20**	0.37	0.59	**0.16**	0.27	0.55
β4	Ethnic	**-2.76**	0.37	0.00	**-2.76**	0.37	0.00	**-2.92**	0.51	0.00
β5	Female*Open				**-0.08**	0.53	0.88			
β6	Ethnic*Open							**0.35**	0.74	0.64
N		**896**			**896**			**896**		
R-sq		**0.09**			**0.09**			**0.09**		

In Model 2, we added the interaction term β5_a_ (female*open) to test whether examiners bias on the basis of gender when grading the exams. A negative coefficient would indicate the presence of a negative bias toward women (female examinees receive lower grades when exams are open compared to blind). Conversely, a positive coefficient would indicate a positive bias towards women (female examinees received higher grades when exams are open compared to blind). The coefficient is small and highly insignificant, however. The analysis does not support the hypothesis that examiners are influenced by knowledge of gender identity when grading exams.

In Model 3, we test whether examiners’ perceptions of examinee ethnic identity matter to their grading. The coefficient for the interaction term β5_b_ (ethnic*open) would indicate a slight positive bias toward ethnic minorities in the sample, but the high p-value suggests that it is far from significant. Thus, the analysis does not support the hypothesis that examiners discriminated against students with names belonging to an ethnic minority in 2018 when examiners could see their names.

As mentioned above, the coders assigned different values to a small percentage of the cases. Discrepancy in the codes is present for 4.1% of the ethnic and 7.2% of the gender identity. To rule out the possibility that this discrepancy might explain the lack of significant bias, we repeated the analyses reported in Table 2 on the sub-sample of cases where all three coders assigned the same code. In both analyses, the interaction terms are highly insignificant, and the conclusion regarding an absence of bias thus holds.

However, what we see at the aggregate level does not rule out the presence of individual-level bias. The aggregate finding is consistent with a situation of underlying contradictory biases in which some examiners bias against females and/or ethnic minorities while others bias in favor of females and/or ethnic minorities. The fact that these might cancel each other out on an aggregate level would matter little to the individual ethnic minority or female examinee subject to negative bias from certain examiners. We therefore analyzed whether different examiners graded differently. In particular, we first analyzed whether examiner gender affected the grading, as hypothesized by Jansson and Tyrefors [19]. As the examiners all belonged to the ethnic majority, we could not test whether ethnicity had any impact.

In Table 3, we analyze gender bias in grading for female and male examiners separately.

**Table 3:** Examiner’s gender and grading bias.

OLS Regression		Model 1			Model 2			Model 3		
		*Basic model*			*Gender bias*			*Ethnic bias*		
**Male examiners**		**B**	SE	P value	**B**	SE	P value	**B**	SE	P value
β0	Intercept	**4.51**	0.43	0.00	**4.32**	0.47	0.00	**4.52**	0.44	0.00
β1	Open	**-0.99**	0.31	0.00	**-0.65**	0.45	0.15	**-1.00**	0.33	0.00
β2	Ordinary exam	**1.00**	0.38	0.01	**1.00**	0.38	0.01	**1.00**	0.38	0.01
β3	Female	**0.37**	0.31	0.23	**0.69**	0.44	0.12	**0.37**	0.31	0.23
β4	Ethnic	**-2.45**	0.43	0.00	**-2.45**	0.43	0.00	**-2.51**	0.62	0.00
β5	Female*Open				**-0.62**	0.61	0.31			
β6	Ethnic*Open							**0.11**	0.86	0.90
N		**666**			**666**			**666**		
R-sq		**0.08**			**0.08**			**0.08**		
**Female examiners**										
β0	Intercept	**4.05**	0.75	0.01	**4.23**	0.77	0.00	**4.03**	0.77	0.00
β1	Open	**-1.50**	0.58	0.01	**-2.22**	0.83	0.01	**-1.47**	0.61	0.02
β2	Ordinary exam	**2.66**	0.80	0.00	**2.76**	0.81	0.00	**2.67**	0.81	0.00
β3	Female	**-0.61**	0.54	0.26	**-1.12**	0.68	0.10	**-0.62**	0.54	0.26
β4	Ethnic	**-3.64**	0.75	0.00	**-3.62**	0.75	0.00	**-3.59**	0.88	0.00
β5	Female*Open				**1.35**	1.11	0.22			
β6	Ethnic*Open							**-0.22**	1.68	0.90
N		**232**			**232**			**232**		
R-sq		**0.15**			**0.16**			**0.15**		

The analysis in Table 3 reveals that examiner gender plays no role for gender bias in exam grading. The interaction term—female*open—is insignificant in both analyses (Model 2). However, the sign of the interaction term differs between the two analyses: The male examiner analysis shows that the coefficient of the interaction term is negative, possibly suggesting that the male examiners grade female examinees lower in the open exam than the blind exam. The female examiner analysis shows that the interaction term is positive, which indicates positive bias toward female students; however, neither result is significant.

The analysis in Table 3 also shows that examiner gender plays no role for introducing ethnic bias in grading, as the interaction term is not significant. The lack of bias in the grading of exams taken by students with ethnic minority names applies equally to male and female examiners.

### Sensitivity analyses

We conducted several supplementary analyses as a robustness check on the results. First, we tested for individual examiner effects. An overall lack of bias can disguise individual-level phenomena. If individual examiners were to show (dis)favor in contradictory ways, the effects disappear at the aggregate level. Only three examiners, all male, graded exams in both 2018 and 2019. We included dummy variables for each of them in the analysis but found no significant bias for any of them. We also repeated the analyses on the sub-sample of cases graded by the three examiners who graded exams both years. The n of this analysis is 458, as the 440 exams graded by teachers only involved in grading either in 2018 or 2019 were excluded.

The results confirmed the findings obtained with the full sample: neither interaction term (gender*open, ethnicity*open) is significant (see S3 Table). Moreover, we ran analyses with robust standard errors as well as a 1,000 bootstrap sample (see S1 and S2 Tables). The results did not deviate from those reported above.

Finally and importantly, we ran an ordered logistic regression. The grading scale is not equidistant, as described in the introduction. At the bottom and around the center of the scale, one step on the scale equals 3 points (-3 to 0; 4 to 7; 7–10); at other intervals of the scale only 2 points (0‒2, 2‒4, and 10‒12). It is therefore necessary to run an ordered logistic regression where the equidistance of the ordinal variable is not assumed. The results of the analysis confirmed the findings reported above. None of the interaction terms used to detect gender or ethnic bias were significant (see S4 Table).

## Discussion

The analysis found no evidence of ethnic or gender bias in the grading of the close to 900 quantitative methods exams. Moreover, we found little to suggest that examiner gender mattered with respect to bias along ethnic or gender divides. Reporting on these types of “non-findings” is important to avoid publication bias, where studies with statistically significant or “spectacularly” strong findings are more frequently submitted and published than studies finding weak or no evidence, which ultimately could risk overstating the evidence for grade discrimination [23]. At the same time, it must be acknowledged that results obtained in one country will always be subject to limitations regarding the scope of generalizations. These pertain to both the Danish context, the specific university culture, as well as the exam format. Moreover, as with most other “natural” experiments, this research design has limitations. While our assumption of constant relative differences in qualifications from one cohort of students to the next appears valid based on similar admissions procedures and admissions data, such simple comparisons might not capture all of the potential sources of relative bias between cohorts. Likewise, any changes to either exam or course structure from one year to the next might disproportionally impact different social groups; for example, ethnicity and gender might affect the extent to which students are likely to take advantage of the new study opportunities (study cafés) introduced in 2019. As this initiative was voluntary, we have no data on who participated, but a potential source of bias would be if ethnic minorities have made less use of this initiative. Another source of bias is the fact that the study only had seven examiners (five in 2018 with open grading). While the limited number of examiners certainly inhibits the scope for generalization, we do believe that the robustness of the results combined with the broadly representative nature of the examiners provides some broader relevance. First, and perhaps most importantly, we found no bias “on average” or at the individual or group (gender) levels, as discussed in the section on sensitivity analyses. Second, the examiners appear to be representative of the broader social science faculty extending beyond RU. The examiners had obtained their degrees and had work experience from other Danish universities before their employment at Roskilde. Of the five examiners who graded exams in 2018, three graduated from and later did doctoral research at other Danish universities (Aarhus, Copenhagen, and Aalborg) before coming to Roskilde. One had just completed doctoral research at Roskilde but had both undergraduate and graduate degrees from the University of Copenhagen. The only RU graduate was an external associate professor (a person who typically holds another job while undertaking certain teaching responsibilities at RU). Moreover, the examiners work in very different fields of research and are therefore unlikely to represent a particular academic culture associated with distinct disciplines or universities.

This study appears to be aligned with many other studies, particularly from Western Europe, which find limited evidence of ethnic or gender bias in grading [9, 12–16]. The rather weak body of evidence of discrimination in grading stands in stark contrast to the much more convincing evidence of ethnic and gender, name-based discrimination in employment, financial, and housing markets. Experimental studies where identical job applications have been submitted under different names belonging to ethnic minorities and majorities provide strong evidence of biases against ethnic minorities [1–3, 5, 6]. This ethnic bias in access to employment interviews also holds true in Scandinavia [4, 7, 8]. Likewise, there is overwhelming evidence that the housing market appears to discriminate against ethnic minorities and males [24, 25]. Again, these studies are primarily based on experimental designs whereby identical rental inquiries are sent to both public and private landlords but using different names. This type of discrimination also appears prevalent in Finland and Sweden [26, 27]. Actors in the financial sector also appear to exhibit biases against ethnic minorities with respect to providing information on investments and loans as well as access to credit [28, 29]. A recent experimental study has documented ethnic discrimination in the provision of public services in Denmark. Identical inquiries were sent out to primary schools from a father asking whether it was possible for his child to move to the school. The father’s name was randomly assigned a typical Danish name or a typical Muslim name. Not only was the acceptance rate significantly lower for inquiries from the supposed Muslim father but they were also faced with greater administrative hurdles in the form of more follow up questions and requests for personal interviews [30].

The fact that studies of grading bias generally find much less evidence of discrimination compared to studies of ethnic biases in public service provisions, employment and housing decisions leads us to forward two tentative explanations that invite further (qualitative) studies capable of illuminating the motivations and dynamics underlying these (quasi)-experimental findings. The first relates to the role of asymmetric information in discrimination [cf. 31]. In grading, examiners have all the relevant information in the form of the actual assignment to make a grading decision. With employment, financial, and housing decisions, decision-makers face asymmetric information, which contributes to statistical discrimination based on stereotyping as a screening mechanism. Related to this, our second explanation is that discrimination depends on the stakes involved in the decision. Not only do decision-makers in the employment, financial, and housing markets face situations characterized by asymmetric information, which provides a greater propensity for speculation and stereotyping, the stakes involved in making wrong decisions are also higher. Contrast this to grading, where the stakes are fairly low; there are no immediate repercussions of grading too low or too high for the decision-maker or the organization as a whole other than the occasional student complaint. The immediate consequences of wrong decisions together with the transaction costs involved in rectifying wrong decisions are usually much higher in the financial, employment, and housing markets. These higher stakes push risk-adverse decision-makers to statistically discriminate based on stereotyping as a means to minimize the risks involved in the decisions. Finally, the variability in discrimination suggests that conscious personal prejudices about ethnicity and genders, while they surely do exist, are less likely to form the basis for discriminatory practices in general. Conscious sexist or race-related beliefs among decision-makers would dictate uniform discriminatory practices across different markets. In fact, the practice of grading provides ample opportunity—due to low levels of competition and oversight—to exercise any conscious sexist and/or racial prejudices that examiners might have. The fact that different “markets” (grading, employment, financing, housing) yield diverse evidence regarding conscious and unconscious biases suggests that discrimination is partly market-specific and dependent on the information available and the stakes involved in the decision.

## Supporting information

S1 Table OLS regression with robust standard errors. (DOCX)

S2 Table OLS regression with bootstrapping. (DOCX)

S3 Table OLS regression with sub sample (graded by examiners who graded both years). (DOCX)

S4 Table Ordered logistic regression. (DOCX)

S1 Data (SAV)
