# Supplementary material for: A quasi-experimental study of ethnic and gender bias in university grading
Source: PLoS One. 2021 Jul 22;16(7):e0254422. doi: 10.1371/journal.pone.0254422 (PMC8297848; doi:10.1371/journal.pone.0254422)
Supplement: S2 Table — (DOCX) [file pone.0254422.s002.docx]

| **S2 Table. OLS regression with bootstrapping** | | | | | | | | | | |
| --- | --- | --- | --- | --- | --- | --- | --- | --- | --- | --- |
|  |  | **Model 1** | | | **Model 2** | | | **Model 3** | | |
|  |  |  | ***Basic model*** |  |  | ***Gender Bias*** |  |  | ***Ethnic bias*** |  |
|  |  | **B** | SE | P value | **B** | SE | P value | **B** | SE | P value |
| β0 | Intercept | **4.48** | 0.36 | 0.00 | **4.46** | 0.39 | 0.00 | **4.51** | 0.37 | 0.00 |
| β1 | Open | **-1.02** | 0.26 | 0.00 | **-0.98** | 0.39 | 0.01 | **-1.07** | 0.30 | 0.00 |
| β2 | Ordinary exam | **1.27** | 0.32 | 0.00 | **1.27** | 0.32 | 0.00 | **1.26** | 0.32 | 0.00 |
| β3 | Female | **0.16** | 0.27 | 0.56 | **0.20** | 0.35 | 0.57 | **0.16** | 0.27 | 0.57 |
| β4 | Ethnic | **-2.76** | 0.31 | 0.00 | **-2.76** | 0.31 | 0.00 | **-2.92** | 0.43 | 0.00 |
| β5 | Female*Open |  |  |  | **-0.08** | 0.52 | 0.89 |  |  |  |
| β6 | Ethnic*Open |  |  |  |  |  |  | **0.35** | 0.62 | 0.57 |
| N |  | **896** |  |  | **896** |  |  | **896** |  |  |
| R-sq |  | **0.09** |  |  | **0.09** |  |  | **0.09** |  |  |
