# Supplementary material for: A quasi-experimental study of ethnic and gender bias in university grading
Source: PLoS One. 2021 Jul 22;16(7):e0254422. doi: 10.1371/journal.pone.0254422 (PMC8297848; doi:10.1371/journal.pone.0254422)
Supplement: S3 Table — (DOCX) [file pone.0254422.s003.docx]

| **S3 Table. OLS regression with sub sample (graded by examiners who graded both years)** | | | | | | | | | | |
| --- | --- | --- | --- | --- | --- | --- | --- | --- | --- | --- |
|  |  | **Model 1** | | | **Model 2** | | | **Model 3** | | |
|  |  |  | ***Basic model*** |  |  | ***Gender Bias*** |  |  | ***Ethnic bias*** |  |
|  |  | **B** | SE | P value | **B** | SE | P value | **B** | SE | P value |
| β0 | Intercept | **4.30** | 0.46 | 0.00 | **4.18** | 0.50 | 0.00 | **4.38** | 0.47 | 0.00 |
| β1 | Open | **-1.00** | 0.37 | 0.01 | **-0.75** | 0.53 | 0.16 | **-1.16** | 0.40 | 0.00 |
| β2 | Ordinary exam | **1.16** | 0.40 | 0.00 | **1.16** | 0.40 | 0.00 | **1.15** | 0.40 | 0.01 |
| β3 | Female | **0.64** | 0.36 | 0.08 | **0.88** | 0.51 | 0.09 | **0.65** | 0.36 | 0.07 |
| β4 | Ethnic | **-2.19** | 0.50 | 0.00 | **-2.20** | 0.51 | 0.00 | **-2.68** | 0.70 | 0.00 |
| β5 | Female*Open |  |  |  | **-0.47** | 0.73 | 0.52 |  |  |  |
| β6 | Ethnic*Open |  |  |  |  |  |  | **1.04** | 1.00 | 0.30 |
| N |  | **458** |  |  | **458** |  |  | **458** |  |  |
| R-sq |  | **0.09** |  |  | **0.09** |  |  | **0.09** |  |  |
