# Supplementary material for: A quasi-experimental study of ethnic and gender bias in university grading
Source: PLoS One. 2021 Jul 22;16(7):e0254422. doi: 10.1371/journal.pone.0254422 (PMC8297848; doi:10.1371/journal.pone.0254422)
Supplement: S4 Table — (DOCX) [file pone.0254422.s004.docx]

| **S4 Table.** | **Ordered Logistic Regression** | | | | | |
| --- | --- | --- | --- | --- | --- | --- |
|  | **Model 1** | | | **Model 2** | | |
|  | **Gender bias** | | | **Ethnic bias** | | |
|  | **B** | Std. Error | p value | **B** | Std. Error | p value |
| Open | **-0.47** | 0.176 | 0.01 | **-0.49** | 0.13 | 0.00 |
| Ordinary | **0.59** | 0.156 | 0.00 | **0.59** | 0.16 | 0.00 |
| Ethnic | **-1.29** | 0.182 | 0.00 | **-1.32** | 0.24 | 0.00 |
| Female | **0.05** | 0.166 | 0.75 | **0.05** | 0.12 | 0.68 |
| Open*Female | **-0.01** | 0.24 | 0.98 |  |  |  |
| Open*Ethnic |  |  |  | **0.08** | 0.36 | 0.82 |
| Threshold |  |  |  |  |  |  |
| -3 | **-6.23** | 0.73 | 0.00 | **-6.24** | 0.72 | 0.00 |
| 0 | **-0.79** | 0.18 | 0.00 | **-0.80** | 0.18 | 0.00 |
| 2 | **-0.25** | 0.18 | 0.17 | **-0.26** | 0.17 | 0.14 |
| 4 | **0.39** | 0.18 | 0.03 | **0.38** | 0.17 | 0.03 |
| 7 | **1.40** | 0.19 | 0.00 | **1.39** | 0.18 | 0.00 |
| 10 | **2.63** | 0.21 | 0.00 | **2.63** | 0.20 | 0.00 |
